# Young male with syphilitic cerebral arteritis presents with signs of acute progressive stroke: A case report

**DOI:** 10.1097/MD.0000000000018147

**Published:** 2019-11-27

**Authors:** Min Shi, Yuan Zhou, Yadi Li, Yuting Zhu, Bing Yang, Li Zhong, Rui Pan, Dongdong Yang

**Affiliations:** a Hospital of Chengdu University of Traditional Chinese Medicine; b Chengdu University of Traditional Chinese Medicine, Chengdu, Sichuan Province, PR China.

**Keywords:** early syphilis, heubner arteritis, mural thrombus, neurosyphilis, progressive ischemic stroke

## Abstract

**Introduction:**

Neurosyphilis is a chronic infection of the central nervous system that is commonly found in adult with long latency periods. Neurosyphilis-attributed deaths in young patients have grown exponentially in the past decade, yet there have been few studies on the early stages of neurosyphilis.

**Patient concerns:**

A young male patient with syphilitic cerebral arteritis was evaluated in our clinic for the clinical signs of progressive ischemic stroke.

**Diagnosis:**

The progression of syphilitic cerebral arteritis was observed through computed tomography imaging, magnetic resonance imaging, magnetic resonance angiogram, and transcranial color Doppler. The pathological changes and clinical outcomes were reviewed. In this specific case, the development of syphilitic cerebral arteritis was dynamic, continuous, and rapid. The pathogenesis was related to Heubner arteritis, in which the formation of a mural thrombus (MT) causes the severe obstruction of blood flow without complete occlusion, leading to an increased risk of infarction. In this patient, formation of the MT resulted in the infarction of the smaller vessels and narrowing of the larger vessels. The partial dislodgment of the MT from the arterial wall of the larger vessels occluded the smaller vessels, leading to infarction.

**Interventions:**

Standard pharmacotherapy for the treatment of the cerebral infarction and a single course of penicillin were applied.

**Outcomes:**

Muscle strength was recovered. The Glasgow Coma Scale score was 15, whereas the NIH Stroke Scale score was 0. The increase in blood flow of the right MCA was accompanied by severe stenosis with compensation of the anterior communicating artery. In addition, moderate to severe stenosis of the right vertebral artery and the basilar artery was suspected. There was a possibility that the right posterior communicating artery was recruited for compensation.

**Conclusion:**

Progressive stroke was the initial symptom of the neurosyphilis. Disease progression is rapid and difficult to control with a single course of penicillin.

## 1 Introduction

Neurosyphilis is a chronic infection of the central nervous system (CNS). Due to the lack of specific symptoms associated with the disease, neurosyphilis is challenging to diagnose.^[1]^ The disease is caused by the invasion of a bacterium known as *Treponema pallidum* into the CNS. The bacterium may lie dormant in patients for several decades before advancing to the rare form of neurosyphilis.^[2]^ According to World Health Organization (WHO), there are approximately 12 million new incidences of syphilis diagnosed every year,^[3]^ and the prevalence of syphilis is on the rise in East Asian countries, such as China.^[4]^ Neurosyphilis-associated vasculitis is often found in older patients with tertiary stage disease who had long latency periods.^[5]^ There have been very few studies on syphilitic cerebral vasculitis, also known as early-stage neurosyphilis, in young patients ranging from 15 to 44 years old. However, neurosyphilis-attributed deaths have grown exponentially in young patients during the past decade.

A direct link between syphilitic meningoencephalitis and stroke was discovered several decades ago.^[6]^ Patients presented with the early signs of progressive stroke, followed by rapid deteriorations in their conditions. Despite treatment with effective antibiotics, the disease failed to respond positively, and the disease progressed into syphilitic cerebral arteritis. Several imaging modalities, including computed tomography (CT), magnetic resonance imaging (MRI), magnetic resonance angiogram (MRA), and transcranial color Doppler (TCD) were performed to assess the course and progression of the disease.

## 2 Case report

This study was approved by the Ethics Committee of the Hospital of Chengdu University of Traditional Chinese Medicine. All procedures performed in studies involving human participants were in accordance with the ethical standards of the institutional and national research committee and with the 1964 Helsinki declaration and its later amendments or comparable ethical standards. Written informed consent was obtained from the individual participant included in the study.

**Figure 1:**
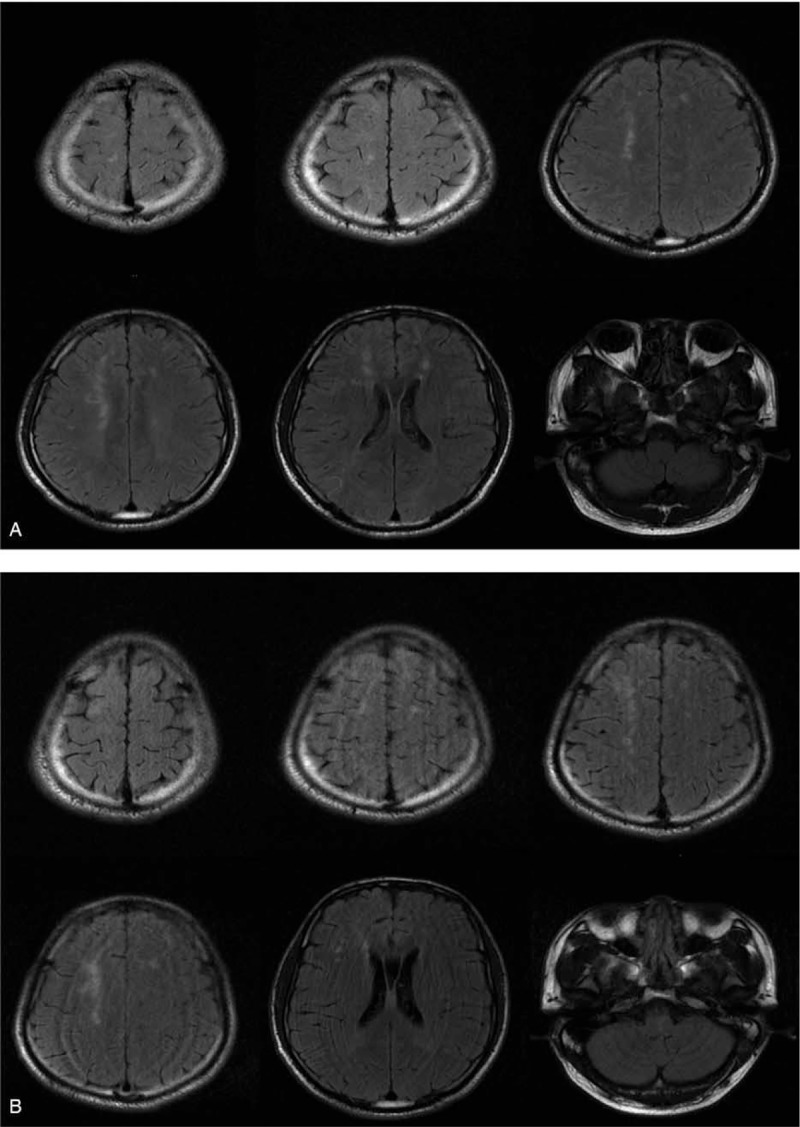
Magnetic resonance imaging (MRI) of a young male with neurosyphilis. MRI revealed (A) multiple plaques of equal-length T2 signal in the bilateral frontal lobes, centrum semiovale, and lateral ventricle, along with hyperintense FLAIR signals, and (B) numerous spots and plaques of equal-length T2 signal in the bilateral frontal lobes, centrum semiovale, right lateral ventricle, and pons, along with hyper-intense FLAIR signals.

**Figure 2:**
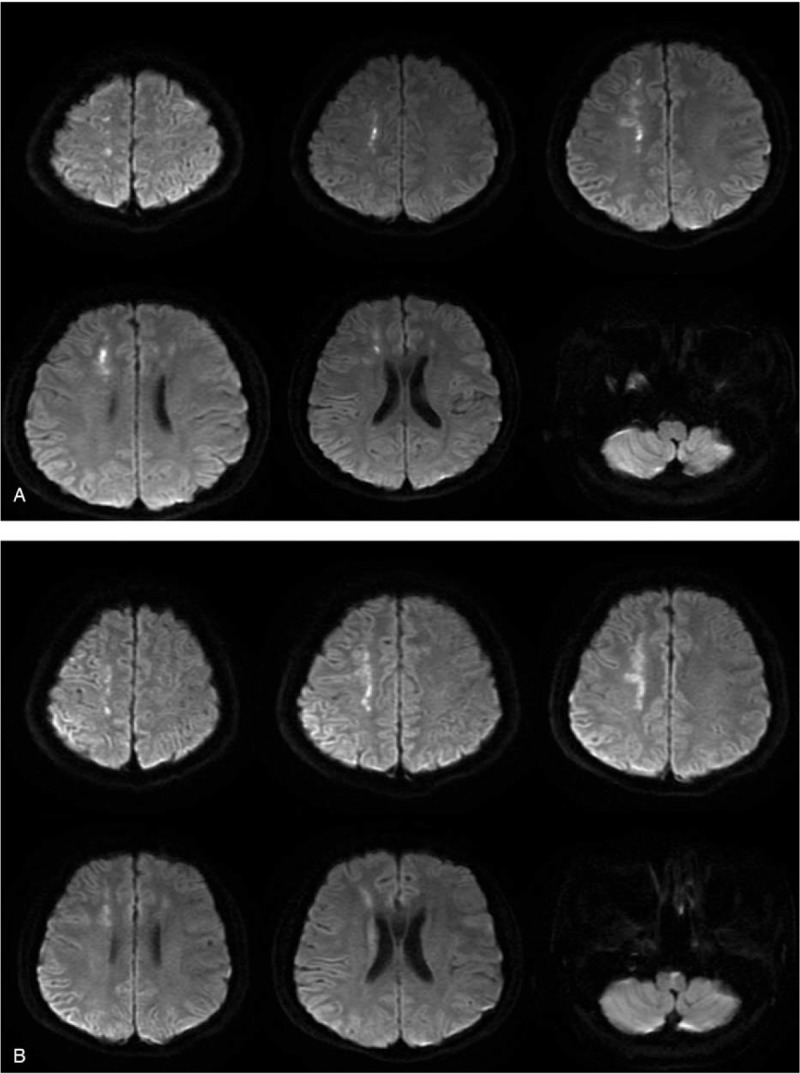
MRI indicated (A) hyper-intense DWI signals of the central lesion in the right centrum and (B) hyperintense DWI signals of lesions in the bilateral centrum semiovale and right lateral ventricle with slightly elevated DWI signals of lesions in the pons.

A 31-year-old male smoker (40 cigarettes/day for 15 years) with no history of hypertension, hyperlipidemia, diabetes, or cardiovascular diseases reported to the clinic with claims of numbness and weakness of the whole body and stumbling on the left side. During the medical interview, the patient admitted to having sexual intercourse with a prostitute 2 months prior. MRI was performed in the Department of Neurosurgery of the Hospital of Chengdu University of Traditional Chinese Medicine. The MRI revealed several plaques in the bilateral ventricles, frontal and parietal lobes, bilateral centrum semiovale, and the right temporal lobe. These small ischemic infarction lesions resulted in demyelination. A new lesion in the right centrum semiovale was detected (Fig. 1A, Fig. 2A). Brain CT imaging revealed small hypointense plaques in the bilateral basal ganglia regions and the side of the lateral ventricle. The patient was later referred to the Department of Neurology outpatient center at the Teaching Hospital of Chengdu University of TCM. The initial diagnosis was idiopathic cerebral infarction and intracranial artery stenosis.

Upon neurological examination, consciousness, verbal responses, long and short memory, directional awareness, as well as the ability to reason, and calculate were normal. The patient's speech was impaired with a hoarse voice. The muscle strength test of the upper left, lower left, upper right, and lower right limbs were level 4+, 3+, 3 and 3-, respectively. Superficial and deep sensations of all of the limbs were abnormal. In addition, Brisk reflexes were observed in the limb tendons. Bilateral Babinski sign (+), bilateral patellar clonus (+), and ankle clonus (+). Scores from the Glasgow Coma Scale (GCS) and National Institutes of Health (NIH) Stroke Scale (NIHSS) were 15 and 4, respectively.

**Figure 3:**
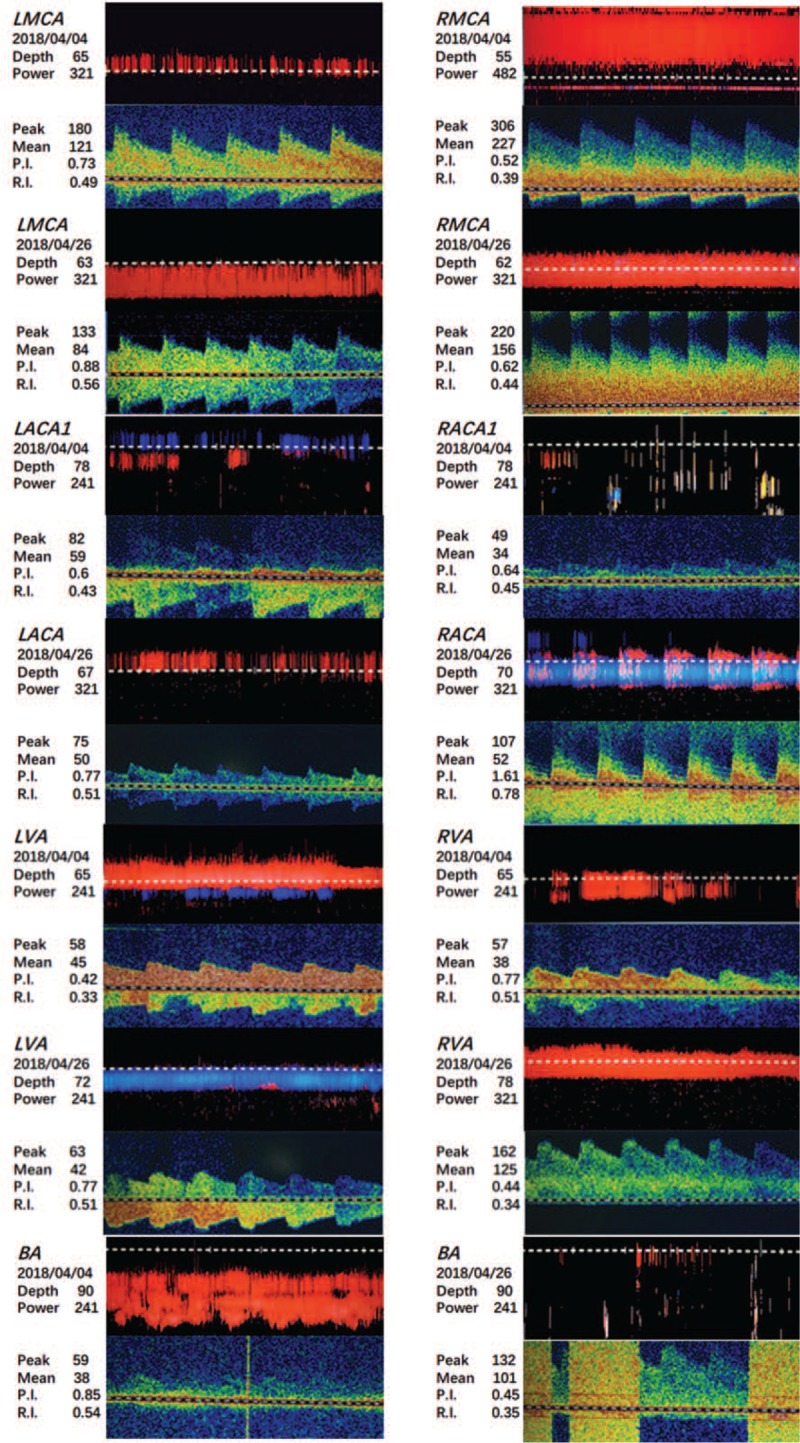
Comparison of TCD images of different blood vessels obtained on April 4, 2018, and April 26, 2018 (Depth: vessel depth; Peak: peak blood flow velocity; Mean: mean blood flow velocity; P.I.: pulsatility index; R.I.: resistance index).

TCD of the eyes and temporalis indicated that presence of increased local blood flow in the bilateral middle cerebral arteries (MCA) with spectral changes. In addition, examination through the eye sockets demonstrated asymmetric blood flow in the bilateral anterior cerebral arteries (ACA1). Decreased blood flow was found in the right ACA1 with the possibility of stenosis. As shown in Figure 3, there were no signs of open anterior or posterior communicating arteries with the Queckenstedt maneuver.^[7]^ The 24 hours dynamic electrocardiograph, color Doppler flow imaging of the carotid artery and vertebrobasilar artery, cranial MRI, and MRA were also performed, along with several laboratory examinations (Table 1).

**Table 1:**
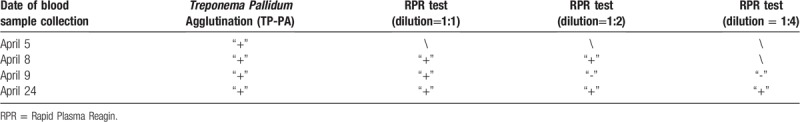
Serum TP-PA and RPR findings from the patient blood sample.

Standard pharmacotherapy for the treatment of the cerebral infarction was applied. Clopidogrel hydrogen sulfate tablets (Hangzhou Sanofi Minsheng Pharmaceutical Co., LTD, 75 mg tablet/day × 7 tablets) were given orally to resist platelet aggregation. In addition, Atorvastatin tablets (Dalian Pfizer, 20 mg tablet/day × 7 tablets) were taken orally to regulate the lipids and stabilize the plaques, Edaravone (Kunming Jida Pharmaceutical, 30 mg/vial) was intravenously injected every 12 hours to protect the nerves, Butylphthalide soft capsules (CSPC NBP Pharmaceuticals, 0.1 g/capsule × 24 capsules) was delivered orally 3 times per day at a dose of 0.2 g each to improve cerebral circulation.

The patient reported that the numbness subsided after treatment. However, the right side of the body still showed signs of weakness. Pathological assessment reported a positive result for the presence of Serodia Treponema pallidum particle agglutination (TP-PA). Skin pruritus was found around the left neck and right palm. The initial diagnosis was eczema. The dermatologist discovered no extraordinary findings while examining the penis and scrotum. A toluidine red unheated serum test (TRUST) was advised before the next follow-up.

**Figure 4:**
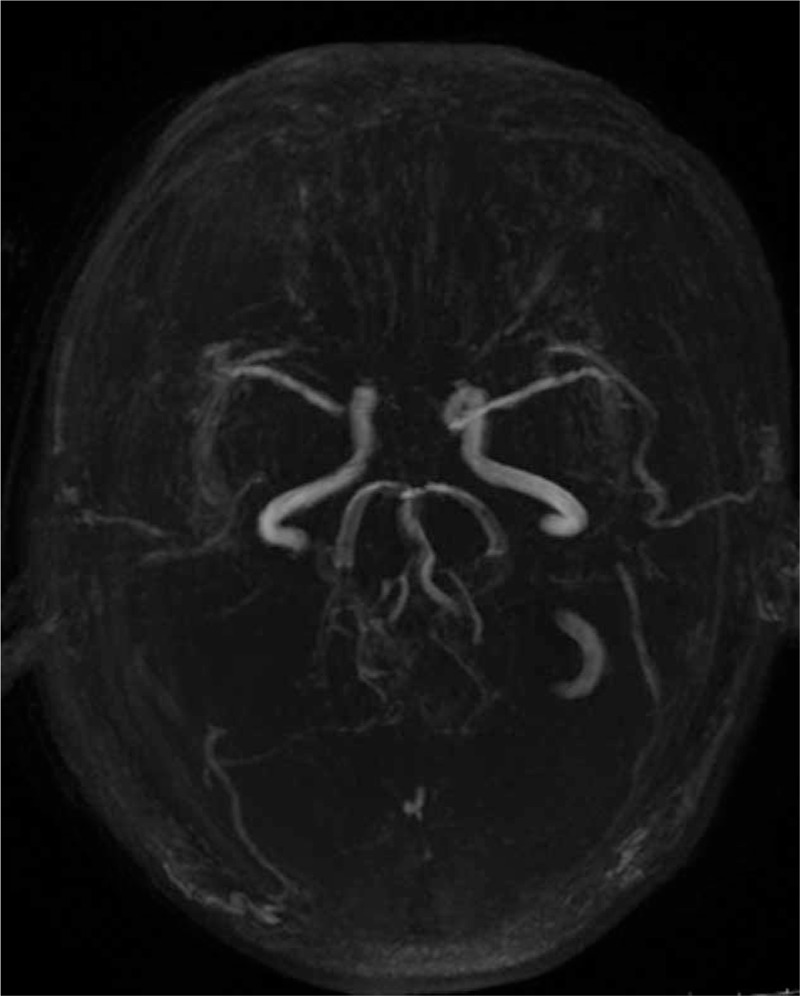
MRA of the young male with neurosyphilis. Bilateral ACA were not revealed, whereas the right MCA was not clearly visualized.

Cranial MRI and MRA showed the presence of multiple small lesions of ischemic infarcts in the bilateral frontal lobes, centrum semiovale, right lateral ventricle, and pons. Partial new infarcts in centrum semiovale and right lateral ventricle, as well as subacute infarcts in the pons, were found. Bilateral ACA were not revealed, while MRA of the right MCA were not clearly visualized. There was no evidence of angiostenosis or malformed blood vessels in the other cerebral components. The neurosyphilis diagnosis was based on findings from the TP-PA, RPR, pathology, and imaging data (Tables 1 and 2, Fig.4Figs. 1B, 2B, and 4).

**Table 2:**
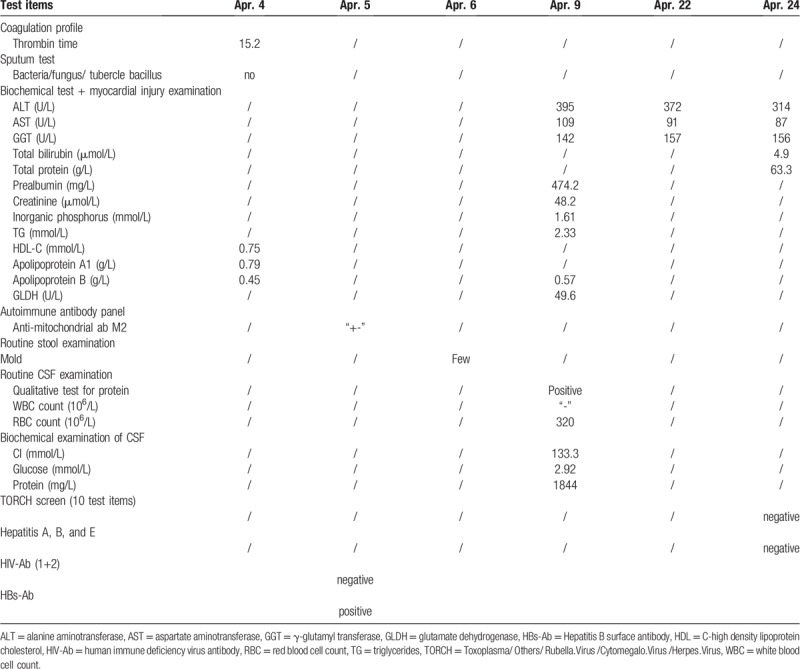
Findings from the biochemical examinations, routine cerebral spine fluid (CSF) examinations, biochemical examinations of the CSF, and other examinations.

The neurosyphilis was treated according to the guidelines from the United States Center for Disease Control, as found in the 2015 Sexually Transmitted Diseases Treatment Guidelines.^[2]^ Treatment included the oral prednisone (Chongqing Kerui Pharmaceuticals, 5 mg/pill × 10 pills) at 0.5 mg/(kg × day) to prevent the Jarisch–Herxheimer reaction. Prednisone was administered to the patient for 3 days before the penicillin therapy. Next, penicillin was administered intravenously (Lukang Pharmaceuticals, 8 × 10^5^ U/vial) at a dose of 3 × 10^6^ U every 4 hours, for 14 consecutive days. As shown in Table 2, alanine aminotransferase (ALT) and aspartate aminotransferase (AST) levels significantly increased during treatment without any apparent symptoms. Clopidogrel hydrogen sulfate tablets, Atorvastatin tablets, Edaravone, and Butylphthalide soft capsule were ceased.

Next, hepatoprotective pharmacotherapy was applied in response to the elevated ALT and AST levels. Polyene phosphatidylcholine (Chengdu Tiantaishan Pharmaceutical Co., 232.5 mg/vial) at a dose of 930 mg/day was delivered intravenously for 2 days followed by an intravenous infusion of compound glycyrrhizin (Eisai China, Inc., 20 ml/vial) and reduced glutathione (Chongqing YaoPharma, 0.6 g/vial) at 1.8 g/day for hepatoprotection. Elevated ALT and AST levels decreased to normal levels by 3 days after starting the treatment (Table 2).

Muscle strength was recovered, and the headache was alleviated after the 14-day penicillin treatment. The muscle strength values of the left upper and lower limbs were 4+ and 3+, while the right upper and lower limbs were 4 and 4−. The Bilateral Babinski sign (+), bilateral patellar clonus (+), and ankle clonus (+). The GCS score was 15, whereas the NIHSS score was 0. The TRUST reexamination indicated a decrease in the dilution value (Table 1).

From the TCD re-examination, it was found that the increase in blood flow of the right MCA was accompanied by severe stenosis of the right MCA with the open anterior communicating artery. In addition, moderate to severe stenosis of the right vertebral artery and the basilar artery was suspected. There was a possibility that the right posterior communicating artery remained open. The hospital failed to contact the patient after discharge, and no follow-up has been conducted from this point onward.

## 3 Discussion

In this case, we have described a young male patient who presented with progressive limb weakness for more than 1 month. Using cranial MRI findings (Figs. 1A, 2A), the patient was initially diagnosed with acute cerebral infarction and the cause remains to be investigated. According to the clinical manifestations, signs, imaging findings (Figs. 3, 1B, 2B, and 4), and laboratory results (Tables 1 and 2), the patient was diagnosed as having progressive cerebral infarction, whereas the diagnosis based on Trial of Org. 10172 in Acute Stroke Treatment (TOAST) classification is syphilitic cerebral arteritis. According to the MRA and TCD findings, the affected vessels developed from the bilateral anterior cerebral arteries, gradually progressed to the bilateral middle cerebral arteries, and eventually to the vertebral and basilar artery. The development of the disease was dynamic and continuous.

The pathological characteristic of neurosyphilis is the invasive thickening of meninges and blood vessels. Syphilitic cerebral arteritis can be divided into 2 sub-categories, including Huebner arteritis and Nissl arteritis, both of which can lead to angiostenosis and angiemphraxis,^[8,9]^ Huebner arteritis or chronic infectious vasculitis may cause inflammation and fibrosis of the vascular adventitia in medium or large arteries. A previous study suggested an increased risk of thrombosis and infarction upon progressive arteriostenosis over time.^[10]^ In these cases, ACA and MCA were commonly involved, which was followed by the basilar artery.^[11]^ However, MRI, MRA, and TCD findings demonstrated that only the aorta was affected without meninges, indicating that it was likely a single Huebner arteritis.

The formation of mural thrombi can obstruct the blood vessels during the development of Huebner arteritis. This causes the infarction of smaller vessels and the narrowing of larger vessels. Partial deterioration of the mural thrombus on the arterial wall of the larger vessels leads to the sudden occlusion of smaller vessels, and ultimately an infarction. To a certain extent, the narrowing of the large vessels can be relieved. In other literature reports and retrospective analyses, we found similar cases of progressive cerebral infarction caused by syphilitic cerebral arteritis.^[12,13]^

Comparing MRA images (Fig. 4) taken at admission and after the final diagnosis, the disease progressed from the involvement of bilateral ACA and MCA to the right vertebral artery. Comparison of the TCD images (Fig. 3) before and after treatment shows that the stenosis of bilateral MCA was alleviated. However, some other arteries were also involved, such as the basilar artery. This suggests that a single course of pharmacotherapy for the cerebral infarction and a single course of penicillin could not prevent the development of syphilitic vasculitis.

Progressive stroke was the initial symptom of the neurosyphilis. MRI, MRA, and TCD were used to monitor the condition as the disease progressed dynamically. Neurosyphilis-induced Huebner arteritis can affect a wide array of blood vessels. Disease progression is rapid and difficult to control with a single course of penicillin. Hence, the effectiveness of standard treatments for cerebral infarctions should be considered when treating patients with neurosyphilis who presents with signs of a cerebral stroke.

## Author contributions

**Conceptualization:** Min Shi, Yuan Zhou, Dongdong Yang.

**Data curation:** Min Shi, Yuan Zhou, Yadi Li, Yuting Zhu, Bing Yang, Li Zhong, Rui Pan, Dongdong Yang.

**Investigation:** Min Shi, Yuan Zhou, Yadi Li, Yuting Zhu, Rui Pan.

**Project administration:** Dongdong Yang.

**Resources:** Min Shi, Yuting Zhu, Li Zhong.

**Supervision:** Dongdong Yang.

**Validation:** Min Shi, Yuan Zhou, Yadi Li, Yuting Zhu, Bing Yang, Li Zhong, Rui Pan, Dongdong Yang.

**Visualization:** Min Shi, Yuan Zhou, Yadi Li, Yuting Zhu, Bing Yang, Li Zhong, Rui Pan, Dongdong Yang.

**Writing – original draft:** Min Shi, Yuan Zhou, Yadi Li, Yuting Zhu.

**Writing – review & editing:** Min Shi, Yuan Zhou, Yadi Li, Yuting Zhu, Bing Yang, Li Zhong, Rui Pan, Dongdong Yang.
